# Identification of miRNAs in Response to Cold Stress in ‘Chaling’ Common Wild Rice (*Oryza rufipogon* Griff.)

**DOI:** 10.3390/life15121896

**Published:** 2025-12-11

**Authors:** Furong Gao, Jincheng Li, Ye Feng, Xiuwen Xiao, Lingling Han, Yufen Ma, Qiuhong Chen

**Affiliations:** 1 College of Agronomy, Hunan Agricultural University, Changsha 410128, China; gaogaofurong@163.com (F.G.); ljc7230072@163.com (J.L.); fy_9779@163.com (Y.F.); xxwzznks@163.com (X.X.); hll1110504@163.com (L.H.); 15532389806@163.com (Y.M.); 2 Yuelushan Laboratory, Changsha 410128, China

**Keywords:** cold stress, rice, differentially expressed miRNA, first nucleotide bias, high-throughput sequencing, target gene

## Abstract

(1) Background: ‘Chaling’ common wild rice (CLWR), one of the two wild rice populations with the northernmost natural distribution worldwide, exhibits excellent cold tolerance. The role of microRNA (miRNA) in regulating cold tolerance in plants has been reported in some species. However, the miRNA landscape in CLWR remains unexplored. (2) Methods: We assessed cold tolerance in CLWR and the conventional rice variety 9311 at 4 °C, and conducted small RNA sequencing and analysis on eight samples from both CLWR and 9311, before and after cold treatment. (3) Results: All seedlings of CLWR survived after cold treatment and recovery, while all seedlings of 9311 died. After quality control and classification analysis of the small RNA sequences, numerous known and novel microRNAs (miRNAs) were identified. The expression analysis showed 59 differentially expressed miRNAs in CLWR before and after cold treatment, and 19 in 9311, with eight overlapping differentially expressed miRNAs between the two varieties. Target gene prediction for these miRNAs indicated that some predicted target genes, such as *CTB4a* and *GRF4*, are key genes involved in regulating cold tolerance in rice. Additionally, CLWR actively mobilizes more miRNAs and their target genes to resist cold stress than 9311. (4) Conclusions: This study offers new insights into the regulatory mechanisms of cold tolerance in CLWR at the miRNA level, providing a wealth of gene (miRNA) resources for genetic breeding research in rice aimed at enhancing cold tolerance.

## 1. Introduction

Low-temperature stress has always been a threat to rice (*Oryza sativa* L.) production, especially as rice from tropical and subtropical regions expands into high-altitude and high-latitude regions, increasing the risk of cold stress exposure. The damage caused by low-temperature stress in rice mainly occurs during the seedling stage and reproductive stage [1,2,3,4]. To mitigate the effects of such adverse weather conditions on rice production, it is crucial and urgent to develop new cold-tolerant rice varieties. Cold tolerance in rice is a complex genetic trait regulated by multiple genes/quantitative trait loci (QTLs). Currently, only a few major genes regulating rice cold-tolerance, such as *qLTG3-1* (quantitative trait locus for low-temperature germinability on chromosome 3), *COLD1* (chilling tolerant divergence 1), *LTT7* (LOC_Os07g22494), *Ctb1* (cold tolerance at booting stage 1), *CTB4a* (cold tolerance at booting stage 4a) and *HAN1* (“han” termed “chilling” in Chinese), have been cloned. The number of cold-tolerance genes available for breeding remains extremely limited [3,5,6]. The cold tolerance of plants depends on the perception and transduction of cold signals by plant cells. Initially, plant cells sense cold stress through decreased fluidity of the plasma membrane (PM), PM-localized G-protein-coupled receptors, or specific cold sensors, including Ca^2+^ channels, two-component system histidine kinases, specific conformations of proteins and nucleic acids, metabolites, etc.) [7,8]. After plants perceive cold stress, the signal is transmitted from the plasma membrane to the cytoplasm and nucleus. Ca^2+^, reactive oxygen species (ROS), and inositol phosphates act as second messengers that are rapidly generated in plant cells following cold stress sensing. These messengers further modulate intracellular Ca^2+^ levels, ROS levels, and protein phosphorylation states. Sensor proteins perceive these changes, interact with target proteins, and regulate the expression of transcription factors and cold-regulated (COR) genes, thereby enhancing plant cold tolerance [3,9,10].

MicroRNAs (miRNAs), a type of small non-coding RNAs (ncRNAs), are encoded by miRNA genes located on the genome. They mediate the regulation of target gene expression through mechanisms including mRNA degradation, translation inhibition, translation enhancement, or generation of trans-acting small interfering RNAs (ta-siRNAs) [11,12]. MiRNAs play important roles in plant development and stress tolerance. Studies on plant miRNA expression patterns under cold stress were first conducted in *Arabidopsis thaliana*, with subsequent research reported in multiple species, such as poplar (*Populus trichocarpa*), grass (*Brachypodium distachyon*), rice, wheat (*Triticum aestivum*), sugarcane (*Saccharum officinarum*), tomato (*Solanum habrochaites*), soybean (*Glycine max*), and cotton (*Gossypium hirsutum*), among others [13,14]. Identifying the expression patterns of miRNAs under cold stress through high-throughput sequencing, Northern blotting, or qRCR is a key approach to discovering cold stress-related miRNAs in plants [13,15]. One of the earliest reported cold-induced miRNAs is *miR397*. Overexpression of *miR397a* in Arabidopsis enhanced plant cold tolerance, leading to significantly higher expression levels of *CBF2* (C-repeat binding factor 2) after 3 h of cold treatment. Following 48 h of cold exposure, the expression levels of certain *COR* genes and *RD29A* (response-to-Dehydration 29A) in overexpressing plants were higher compared to the wild type. These results suggest that *miR397a* may enhance plant cold tolerance by upregulating downstream *COR* genes via modulation of the *CBF* pathway [16]. Overexpression of *osa-miR156* enhances cold tolerance in transgenic Arabidopsis, rice, and pine (*Pinus elliottii*). In *osa-miR156*-overexpressing rice, the transcript levels of *OsSPL3* (SQUAMOSA promoter binding protein-like) were significantly reduced. *OsSPL3* can bind to the promoter region of *OsWRKY71* to promote its transcription. The decreased expression of *OsSPL3* indirectly reduced *OsWRKY71* levels, which downregulated two key transcription factors, *OsMYB2* (myeloblastosis 2) and *OsMYB3R-2*. *OsLEA3* (late embryogenesis abundant 3), *OsRab16A* (responsive to ABA 16A), and *OsDREB2* (dehydration-responsive element-binding protein 2) are downstream genes of *OsMYB2*. In *osa-miR156*-overexpressing rice, the transcriptional levels of *OsLEA3* were elevated, and cold tolerance was improved [17]. Similarly, *osa-miR528* overexpression enhances cold tolerance in Arabidopsis, pine, and rice [18]. In contrast, *miR1432* negatively regulates rice cold tolerance [19].

The ‘Chaling’ common wild rice (*Oryza rufipogon* Griff.) population is one of the two northernmost wild rice populations globally. By relocating CLWR to Changsha, China, (beyond the natural northern limit of wild rice in Dongxiang, Jiangxi), CLWR roots successfully survived winter for multiple consecutive years [20,21]. From 13 January to 3 February 2008, the daily average temperature in Changsha was −1 °C, with the lowest temperature reaching −4.7 °C. Under these conditions, all CLWR and Dongxiang wild rice roots in the experimental field survived, whereas only partial survival was observed for ‘Fusui’, ‘Jiangyong’, and ‘Liujiang’ wild rice accessions. In contrast, roots of 11 cultivated rice varieties, along with the roots of ‘Hainan’ and ‘Hepu’ wild rice, completely died [20]. These findings demonstrated CLWR’s exceptional cold tolerance. Currently, there are no reports on miRNAs associated with cold stress in CLWR. In this study, seedlings of CLWR and indica rice 9311 (*Oryza sativa* L.) were subjected to cold stress, followed by small RNA sequencing and analysis of samples collected before and after treatment. This study aims to identify miRNAs associated with cold tolerance, provide gene (miRNA) resources for cold-tolerant rice breeding, and analyze the cold tolerance of CLWR at the miRNA level.

## 2. Materials and Methods

### 2.1. Rice Materials

The rice materials used in this experiment were ‘Chaling’ common wild rice (CLWR) and the conventional indica rice variety 9311, using 9311 as the cold-sensitive control. The seedlings of CLWR are slender with a relatively upright plant architecture, while the seedlings of 9311 are robust. The seeds of CLWR were originally collected from their native habitat and have since been propagated annually together with 9311 in the experimental fields of Hunan Agricultural University.

### 2.2. Cold Tolerance Assessment and Sampling

Healthy seeds of CLWR and 9311 were soaked and germinated. Subsequently, they were sown in the same nursery tray with Yoshida nutrient solution (Coolabor Technology Co., Ltd., Beijing, China) and grew in a growth chamber under standard conditions (14 h light at 28 °C/10 h dark at 25 °C) until the three-leaf stage. For cold stress treatment, the chamber conditions were then adjusted to a constant 4 °C with a 14 h photoperiod. After the seedlings were subjected to cold stress for 48 h, growth chamber conditions were restored to the standard conditions (14 h light at 28 °C/10 h dark at 25 °C) for a 7-day recovery period. Before and after the cold stress treatment, the phenotypes of CLWR and 9311 plants were compared. This experiment was conducted with a minimum of five independent biological replicates. Before and after 48 h of cold treatment, whole plant samples of CLWR and 9311 were collected separately. A portion of each sample was sent to Majorbio Bio-Pharm Technology Co., Ltd. (Shanghai, China) for total RNA extraction, small RNA library construction, and sequencing. The remainder was used for RNA extraction to detect the expression levels of miRNAs by qRT-PCR. The eight samples of CLWR and 9311 are listed in Table 1. A represents the sample group of CLWR before cold treatment, including two replicate samples (CLWR1 and CLWR2); B represents the sample group of CLWR after cold treatment for 48 h, including two replicates (CLWR3 and CLWR4); C represents the sample group of 9311 before cold treatment, including two replicates (C9311_1 and C9311_2); D represents the sample group of 9311 after cold treatment for 48 h, including two replicates (C9311_3 and C9311_4).

### 2.3. Small RNA Sequencing and Quality Assessment

The RNA concentrations of the eight samples ranged from 163 ng/μL to 479 ng/μL, and 3 μg of total RNA from each sample was used for library construction. Small RNAs were isolated from the total RNA of each sample, and the small RNA libraries were constructed using the SMARTer smRNA-Seq Kit for Illumina (Clontech, Mountain View, CA, USA). These libraries were then sequenced on the Illumina NovaSeq 6000 platform. Sequencing quality assessment was performed on the raw sequencing data for each sample. Raw reads were filtered on the Majorbio Cloud Platform (https://cloud.majorbio.com/page/project/overview.html, accessed on 18 January 2024) using Fastx-Toolkit (Version 0.0.14) to obtain clean reads and useful reads (clean reads of 18–32 nt). The filtering steps were as follows: (1) Remove the 3′ adapter sequences from the reads, discard reads without inserted fragments. (2) Trim low-quality bases (quality value <20) from the 3′ end of the reads. (3) Discard reads containing unknown bases (‘N’). (4) Discard reads shorter than 18 nt. (5) Discard reads longer than 32 nt).

### 2.4. Small RNA Classification and miRNA Family Analysis

Useful reads were aligned to the rice reference genome (IRGSP-1.0) (http://plants.ensembl.org/Oryza_sativa/Info/Index, accessed on 3 February 2024) using Bowtie2 (Version 2.2.9) to obtain genome-mapped reads [22,23]. These mapped reads were then aligned to the rice miRNA precursors and mature sequences in the miRBase database (Version Release 22) (http://www.mirbase.org/) to identify known miRNAs [24]. The mapped reads that did not match known miRNAs were aligned to the Rfam database (Version Rfam v12.3) (http://rfam.xfam.org/) to identify ncRNAs such as ribosomal RNA (rRNA), transfer RNA (tRNA), small nuclear RNA (snRNA) and small nucleolar RNA (snoRNA) [25]. The remaining mapped reads were further aligned to the rice genome to identify small RNAs originating from repetitive sequences (repbase), as well as to the exonic and intronic regions of mRNA to identify small RNAs derived from mRNA degradation fragments. The remaining unannotated small RNA sequences were aligned to the rice genome. The small RNA and flanking sequences surrounding each alignment were then extracted and used to predict novel miRNAs with miReap (Version 0.2) software [26]. The secondary structure, dicer cleavage sites, minimum free energy, and other characteristics of the predicted novel miRNAs were analyzed. From this analysis, information such as the mature sequence, length, precursor sequence, and energy was determined. For both conserved miRNAs and novel miRNAs, the mature miRNAs derived from the 5′ arm and 3′ arm of the same precursor were labeled with the miRNA name followed by ‘−5p’ and ‘−3p’, respectively. The number of reads for each category of small RNA was counted. All miRNAs were subjected to first nucleotide bias analysis. Based on the miRNA seed sequence, known miRNAs were assigned to families, and the conservation of miRNA families across different species was investigated.

### 2.5. The Expression Analysis of miRNAs

The quantifier script in miRDeep2 (Version 2.0.1.3) software was used to quantify the expression levels of known and novel miRNAs in each sample [27]. The expression levels of miRNAs were measured using TPM (Transcripts Per Million reads) to normalize for miRNA length and sequencing depth. The edgeR (Version 3.24.3) software was used to perform differential expression analysis of miRNAs between samples or groups [28]. Differentially expressed miRNAs were identified using the parameters adjust *p*-value (FDR) < 0.05 and |log_2_FC| ≥1. edgeR normalizes sample libraries based on read counts. Its normalization steps are as follows: (1) Remove genes (miRNAs) with 0 read counts in all samples. (2) Pick one sample to be the “reference sample” for normalizing all the other samples. (3) Using the reference sample, gene sets are created for the remaining samples based on the normalized read counts. In this process, biased genes will be filtered out. (4) Calculate the weighted average of the log_2_ ratios for each sample’s gene set. (5) Convert the weighted average of the log_2_ ratios to a scaling factor using the formula $2^{\left\{weighted\;average\;of\;the\;log2\;ratios\right\}}$, thus obtaining the original normalization factor for each sample. (6) Perform centralization of the original normalization factors, thereby obtaining the final normalization factor for each sample, then divide the values in the expression matrix of each sample by its corresponding normalization factor.

### 2.6. Target Gene Prediction for All Differentially Expressed Known miRNAs

The psRobot (Version v1.2) software was used to predict target genes for all differentially expressed miRNAs [29]. The target genes were functionally annotated using the following six databases: NR (http://ftp.ncbi.nlm.nih.gov/blast/db/, accessed on 5 February 2024), Swiss-Prot (http://web.expasy.org/docs/swiss-prot_guideline.html, accessed on 5 February 2024), EggNOG (http://eggnogdb.embl.de/#/app/home, accessed on 5 February 2024), GO (http://www.geneontology.org), KEGG (http://www.genome.jp/kegg/), and Pfam (http://pfam.xfam.org/) [30,31,32,33,34,35,36]. GO enrichment analysis for the target gene set was carried out using Goatools (Version 0.6.5), with an adjusted *p*-value (*p*-adjust) of <0.05 set as the significance threshold [37].

### 2.7. Expression Level Detections of miRNAs by qRT-PCR

Total RNA was extracted from rice samples using the Trizol reagent (Invitrogen, Carlsbad, CA, USA). The quality and purity of RNA were evaluated using a Nano spectrophotometer (Aosheng Co., Ltd., Hangzhou, China) and agarose (Solarbio Science & Technology Co., Ltd., Beijing, China) gel electrophoresis. Following the method of Chen et al. [38], we detected miRNA expression levels by qRT-PCR using rice *ubiquitin* (Os03g0234200) as the reference gene on the StepOne^®^ Real-Time PCR System (Applied Biosystems, made in Singapore). The raw data of qRT-PCR were acquired using StepOne Software v2.3. The 2^−∆∆Ct^ method was used to calculate relative expression levels [39]. Statistical analysis was performed using DPS 7.5 (Version 7.5), with significance levels indicated as follows: * *p* < 0.05 and ** *p* < 0.01. The primers used for miRNA detection are listed in Supplementary Excel File S1.

## 3. Results

### 3.1. CLWR Exhibited a Strong Cold Tolerance

**Figure 1:**
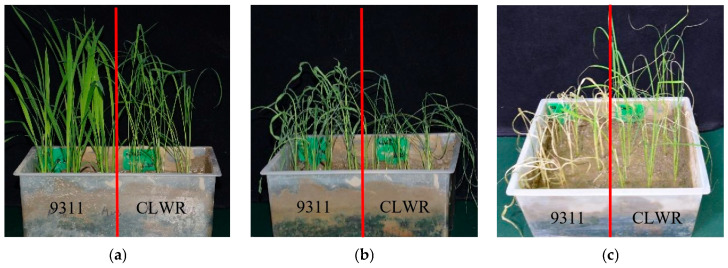
The cold tolerance identification of CLWR and 9311 seedlings. (**a**) Seedlings before cold stress treatment; (**b**) Seedlings after cold stress treatment (4 °C) for 48 h; (**c**) Seedlings normally cultured for 7 days after cold stress treatment; CLWR: ‘Chaling’ common wild rice.

The seedlings of CLWR and 9311 were subjected to cold stress treatment at 4 °C. From the results shown in Figure 1, it could be observed that the seedlings of 9311 exhibited wilting and leaf curling after 48 h of cold stress, and even after 7 days of recovery under normal temperature, all the seedlings died. On the other hand, although the seedlings of CLWR also showed leaf curling after 48 h of cold stress, they all survived after 7 days of recovery.

### 3.2. High-Throughput Sequencing of the Small RNA Transcriptomes of CLWR and 9311 Before and After Cold Treatment

The eight samples of CLWR and 9311 before and after cold treatment in Table 1 were sent to the company for RNA extraction. Eight RNA samples that met the requirement were used to construct small RNA libraries and subjected to high-throughput sequencing. As shown in Table S1, a large number of raw reads, clean reads and useful reads (18 to 32 nt clean reads) were obtained in each sample. The Q20 values (calculated for each sample’s clean reads) of all eight samples were greater than 98%, with seven samples greater than 99%, indicating that the sequencing quality of all samples was qualified. Then all the useful reads were aligned to the rice genome to obtain the mapped reads.

**Figure 2:**
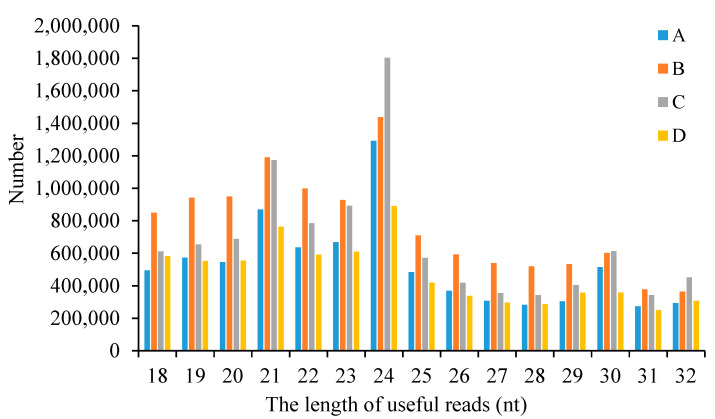
The length distribution of useful reads in four sample groups. A: The group of CLWR before cold treatment; B: The group of CLWR after cold treatment; C: The group of 9311 before cold treatment; D: The group of 9311 after cold treatment.

The length distribution of the useful reads in four sample groups was analyzed. The data for each sample group is the average of the data from two replicate samples. As shown in Figure 2, the length distribution of the useful reads across all four sample groups predominantly ranges from 18 to 24 nt, with a pronounced peak at 24 nt.

### 3.3. Classification of Small RNAs

The mapped reads of each sample were sequentially aligned to identify known miRNA, rRNA, tRNA, snoRNA, snRNA, repbase, mRNA degradation fragment (exon and intron) and novel miRNA. The sequences and other information of all identified known miRNAs and novel miRNAs are displayed in Supplementary Excel Files S2 and S3. The remaining unannotated small RNA sequences were classified as unknown. The number of reads (including duplicate counts) for different types of small RNAs in each sample and their proportions are shown in Table 2. The results indicate that none of the samples contain snoRNA. The largest proportion is unknown reads.

The known miRNAs from the two replicate samples were merged together, and similarly, the novel miRNAs from the two replicate samples were merged together. Duplicate reads were removed, resulting in the number of known miRNA types and novel miRNA types in each sample group. According to the results in Table 3, the number of known miRNAs types in each sample group ranged from 397 to 443, but the numbers of novel miRNAs types across different sample groups showed significant differences. Combining the results of Table 2 and Table 3, it can be observed that although all sample groups have a higher number of novel miRNAs types compared to known miRNAs types (Table 3), the number of reads (including duplicates) for known miRNAs is much higher than that for novel miRNAs in each sample (Table 2), which suggests that the known miRNAs have higher expression levels and more duplicates compared to novel miRNAs.

### 3.4. Length and Nucleotide Preference of Known miRNAs and Novel miRNAs

All known miRNAs and novel miRNAs from Table 3 were integrated together, and duplicate miRNAs were removed. The number of known miRNA types and new miRNA types for different lengths was counted. According to the results in Table 4, the length distribution of known miRNAs ranges from 19 to 25 nt, with the highest number of known miRNAs (827) being 21 nt. The length distribution of novel miRNAs ranges from 18 to 27 nt, with the highest number of novel miRNAs (2149) being 24 nt.

**Figure 3:**
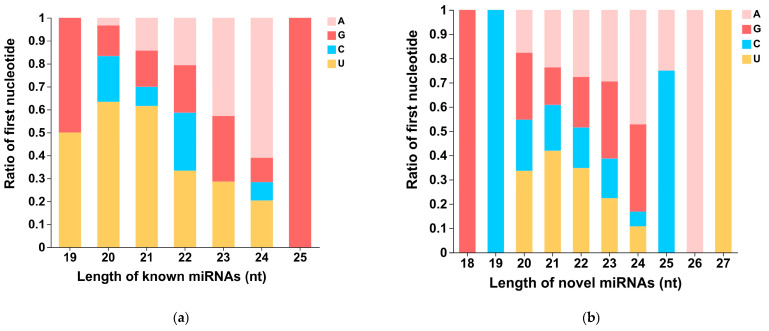
The first nucleotide bias of known miRNAs and novel miRNAs of different lengths. (**a**) The first nucleotide bias of known miRNAs. (**b**) The first nucleotide bias of novel miRNAs. A: Adenylate; G: Guanylate; C: Cytidylate; U: Uridylate.

The first nucleotide bias of known miRNAs and novel miRNAs for different lengths is displayed in Figure 3a and Figure 3b, respectively. Known miRNAs with lengths of 20 nt and 21 nt show a preference for using Uridylate (U) as the first nucleotide, while known miRNAs with a length of 24 nt preferentially use Adenylate (A) as the first nucleotide. Novel miRNAs with lengths of 20 nt, 21 nt, and 22 nt preferentially use Uridylate (U) as the first nucleotide. However, novel miRNAs with a length of 24 nt show a stronger preference for using Adenylate (A) and Guanylate (G) as the first nucleotide. Due to the limited number of known miRNAs with lengths of 19 nt (2) and 25 nt (1), as well as novel miRNAs with lengths of 18 nt (1), 19 nt (1), 26 nt (1), and 27 nt (1), the statistical analyses of nucleotide bias for these lengths of miRNAs are not meaningful.

### 3.5. The Distribution of Known miRNA Families in Different Species

MiRNAs have been found to be widely present in various species, including animals, plants, DNA viruses, and single-celled organisms. In different species, some miRNAs exhibit conservation, while others show specificity [40,41]. In this study, the distribution of 63 identified known miRNA families was analyzed across 60 different species, including animals, plants, and microorganisms. The results in Table 5 show that these miRNA families can be classified into three categories. The first category consists of miRNA families that are exclusively present in plants, such as *MIR156*, *MIR159*/*319*, and *MIR160*, etc. The second category includes miRNA families that are found only in Poaceae plants, such as *MIR1878*, *MIR2275*, *MIR437*, *MIR444*, *MIR529*, and *MIR531*. The last category represents miRNA families that are unique to rice, such as *MIR1319*, *MIR1428*, *MIR1437*, and so on.

### 3.6. The Expression Level of miRNAs in Different Samples and Groups

**Figure 4:**
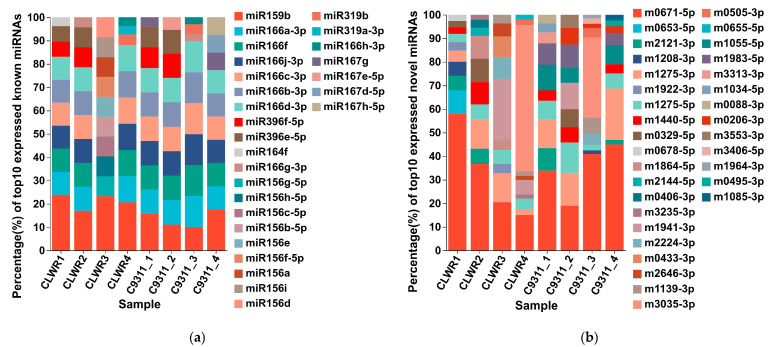
Top 10 known miRNAs and novel miRNAs ranked by expression levels in each sample. (**a**) The top 10 known miRNAs in each sample; (**b**) The top 10 novel miRNAs in each sample. Note: The different colored squares on the right represent different miRNAs. The height percentage of each color column in a sample represents the proportion of expression level for that miRNA among the total expression levels of these 10 miRNAs.

The expression levels based on TPM values of all identified known and novel miRNAs in transcriptomes are shown in Supplementary Excel File S4. Figure 4 displays the top 10 known and novel miRNAs ranked by expression levels in each sample. Among the known miRNAs, *osa-miR159b* exhibits the highest expression level in most samples, followed by *osa-miR166a-3p*. The composition of the top 10 known miRNAs shows minimal variation across all samples. For novel miRNAs, *nov-m0671-5p* has the highest expression level in most samples. However, there are significant variations in the composition of the top 10 novel miRNAs across different samples. PCA (Principal Component Analysis) based on the expression levels of miRNAs for the data from eight samples was also conducted on the online platform of Majorbio Cloud Platform. The results revealed that the clustering between two replicate samples was relatively close (Figure S1), further demonstrating the reliability of both the sampling and sequencing data in this study.

**Figure 5:**
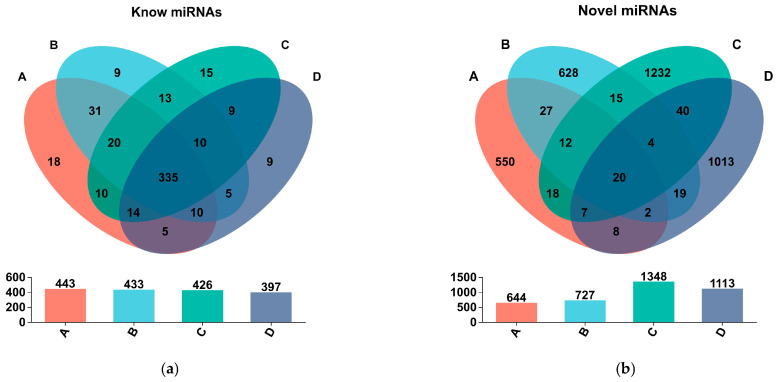
The Venn analysis results of known and novel mature miRNAs among different sample groups. (**a**) Venn analysis of known miRNAs; (**b**) Venn analysis of novel miRNAs. Note: Circles of different colors represent different groups, the numbers represent the numbers of miRNAs, and the numbers in the cross areas represent the number of shared miRNAs among groups.

The known and novel mature miRNAs that were shared among the sample groups or unique to each sample group were obtained by Venn analysis. Figure 5 shows that there are 335 known miRNAs and 20 novel miRNAs expressed in all four sample groups, which may represent the highly conserved miRNAs. Group A and group B share 396 known miRNAs and 61 novel miRNAs. Group C and group D share 368 known miRNAs and 71 novel miRNAs. There are 31 known miRNAs and 27 novel miRNAs that are shared between group A and group B but not present in group C and group D. These miRNAs may be specifically expressed in CLWR. Similarly, there are 9 known miRNAs and 40 novel miRNAs that are shared between group C and group D but not present in group A and group B. These miRNAs may be specifically expressed in 9311. There are 5 known miRNAs and 19 novel miRNAs that are shared between group D and group B but not present in group C and group A, suggesting that these miRNAs may be involved in cold response.

### 3.7. Differentially Expressed miRNAs Before and After Cold Stress in CLWR and 9311

**Figure 6:**
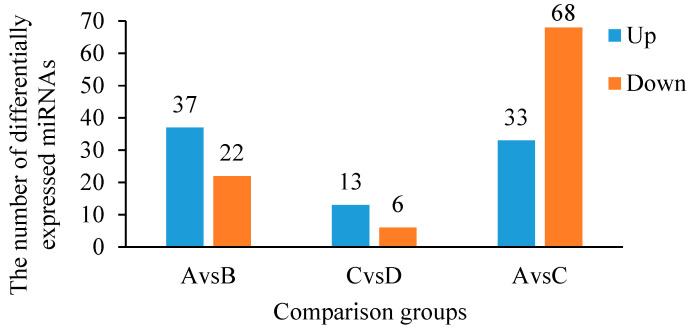
The numbers of differentially expressed miRNAs between each two groups. Note: Group A serves as the control group for the comparison between group A and group B; group C serves as the control group for the comparison between group C and group D; group A serves as the control group for the comparison between group A and group C.

Statistical analyses were conducted on the expression levels of each miRNA in each sample group to identify differentially expressed miRNAs between groups. The expression level of each miRNA in each sample group is represented by the average expression level of the two replicate samples (Supplementary Excel File S4). The results in Figure 6 show that there are 59 differentially expressed miRNAs between group A (CLWR before cold stress treatment) and group B (CLWR after cold stress treatment). Among these, 37 miRNAs show significantly upregulated expression in group B, while 22 miRNAs show significantly downregulated expression. For group C (9311 before cold stress treatment) and group D (9311 after cold stress treatment), there are 19 differentially expressed miRNAs. Among them, 13 miRNAs exhibit significantly upregulated expression in group D, while 6 miRNAs show significantly downregulated expression. In the comparison between group A and group C, there are 101 differentially expressed miRNAs. Among them, 33 miRNAs show significantly upregulated expression in group C, while 68 miRNAs show significantly downregulated expression. The specific names of the differentially expressed miRNAs between group A and group B (AvsB), as well as between group C and group D (CvsD), are displayed in Table 6. MiRNAs marked with two asterisks (**) indicate that these miRNAs exhibit differential expression in both the AvsB and CvsD comparisons.

### 3.8. Validation of the Expression Levels of the miRNAs by qRT-PCR

**Figure 7:**
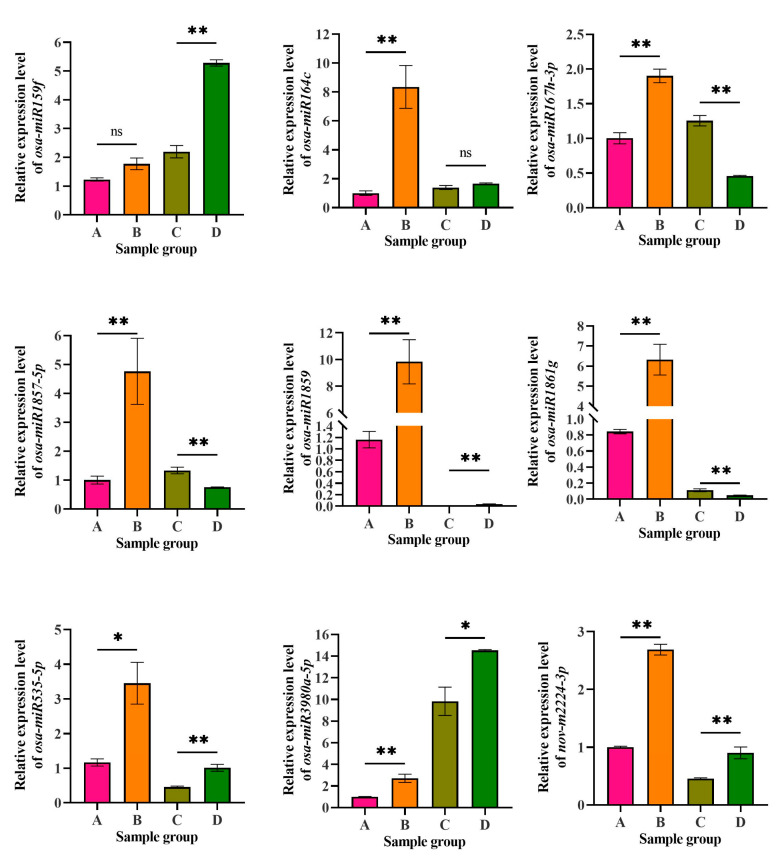
The expression level detection results of nine miRNAs by qRT-PCR. A: CLWR before cold treatment; B: CLWR after 4 °C cold treatment for 48 h; C: 9311 before cold treatment; D: 9311 after 4 °C cold treatment for 48 h. Note: The corresponding accession number of the miRNAs in miRBase: *osa-miR159f* (MIMAT0001027), *osa-miR164c* (MIMAT0001033), *osa-miR167h-3p* (MIMAT0022874), *osa-miR1857-5p* (MIMAT0007779), *osa-miR1859* (MIMAT0007792), *osa-miR1861g* (MIMAT0007802), *osa-miR535-5p* (MIMAT0003142), *osa-miR3980a-5p* (MIMAT0019675), *nov-m2224-3p* (novel miRNA, the mature and precursor sequence information can be found in Supplementary Excel File S3). Rice *ubiquitin* (Os03g0234200) was used as the reference gene. Statistical analysis was performed using DPS 7.5, with significance levels indicated as follows: * *p* < 0.05 and ** *p* < 0.01.

Nine miRNAs were selected for qRT-PCR amplification to validate their relative expression levels in CLWR and 9311 before and after cold treatment. The expression levels of these miRNAs in the small RNA transcriptomes are displayed in Table S2, represented as TPM values. The qRT-PCR results are shown in Figure 7. In CLWR, except for *osa-miR159f*, the expression levels of all tested miRNAs were significantly upregulated after cold treatment. Among them, *osa-miR164c*, *osa-miR1859* and *osa-miR1861g* exhibited a larger fold change. In 9311, although the expression levels of *osa-miR1859* significantly increased after cold treatment, it remained relatively low both before and after the cold treatment. *Osa-miR167h-3p*, *osa-miR1861g* and *osa-miR1857-5p* showed significant downregulation after cold treatment, while *osa-miR159f*, *osa-miR535-5p*, *osa-miR3980a-5p* and *nov-m2224-3p* exhibited significant upregulation after cold treatment. *Osa-miR3980a-5p* showed relatively higher expression level in 9311 compared to CLWR. Overall, except for a few miRNAs, these 9 miRNAs showed a consistent expression pattern in the small RNA transcriptome data compared to the qRT-PCR results, indicating that the statistical analysis of miRNA expression level in the small RNA transcriptome data is reliable.

### 3.9. The Predicted Target Genes and Their Functions for the Differentially Expressed miRNAs

**Figure 8:**
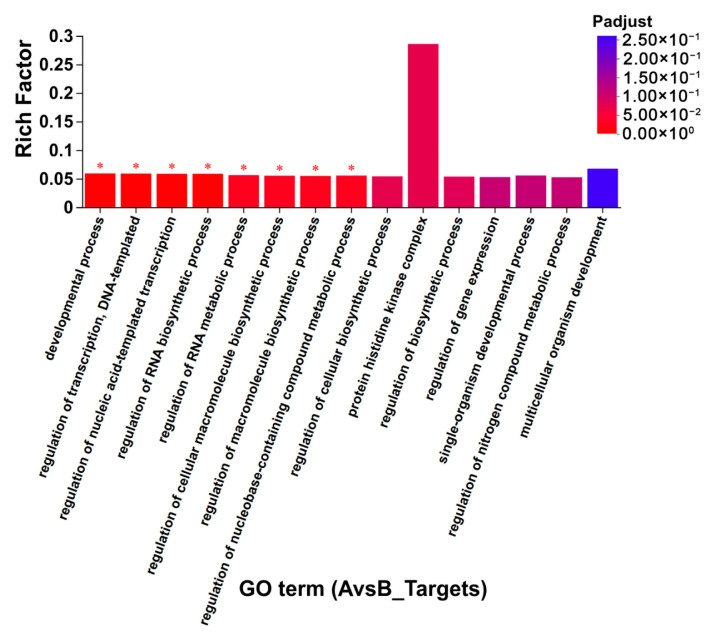
The GO term enrichment results of the targets genes of differentially expressed miRNAs in CLWR. Note: The asterisk (*) indicates a significant enrichment of the target genes in a specific GO term (*p* < 0.05).

Since miRNAs exert their biological functions by regulating the expression of target genes, it is important to identify the target genes of miRNAs to understand their functions. Target gene prediction was performed for differentially expressed miRNAs in CLWR (AvsB, results displayed in Supplementary Excel File S5) and in 9311 (CvsD, results displayed in Supplementary Excel File S6). Some reported genes regulating rice cold-tolerance, such as *CTB4a* (Os04g0132500) and *OsGRF4* (growth-regulating factor 4) (Os02g0701300), were found among these predicted target genes [5,42]. Among the 59 differentially expressed miRNAs in CLWR, 57 miRNAs were predicted to have target genes, with *nov-m2417-3p* having the highest number of target genes (475). Among the 19 differentially expressed miRNAs in 9311, 17 miRNAs were predicted to have target genes, with *nov-m2224-3p* having the highest number of target genes (150). Further GO term clustering and enrichment analyses of the target genes were performed for the differentially expressed miRNAs in CLWR and 9311 separately. The results in Supplementary Excel Files S7 and S8 show that the target genes of differentially expressed miRNAs in CLWR and 9311 exhibit broader GO term clustering, but there are also some differences. Overall, there were more target genes and clustering GO terms identified in CLWR. The results in Figure 8 and Figure S2 show that the target genes of differentially expressed miRNAs in CLWR are significantly enriched in eight GO terms, while the target genes of differentially expressed miRNAs in 9311 do not exhibit significant enrichment in any GO term.

## 4. Discussion

### 4.1. Characteristics of miRNAs in CLWR and 9311

The application of high-throughput sequencing technology has accelerated the identification of small RNAs, especially miRNAs, in various species [43,44,45]. In this study, small RNA transcriptome sequencing was performed on eight samples of CLWR (cold-tolerant rice) and 9311 before and after cold treatment. Following quality control, sequence alignment, and classification, known and novel miRNAs were identified in each sample. Comparative analysis revealed that the number of novel miRNA types was much higher than that of known miRNA (Table 3), aligning with the hypothesis that plants possess a large repertoire of recently evolved miRNAs [41]. However, known miRNAs exhibited much higher expression levels than novel miRNAs (Table 2), consistent with previous findings that known miRNAs are evolutionarily conserved and functionally critical, whereas novel miRNA genes may lack regulatory elements required for stronger expressions and may not be integrated in regulatory networks with essential biological functions [46]. Both known and novel mature miRNAs in this study showed a length distribution mainly ranging from 20 to 24 nt, with 21 nt being the most abundant length for known miRNAs and 24 nt for novel miRNAs (Table 4). The length of mature miRNAs depends on the cleavage of precursors by Dicer-like (DCL) enzymes. Specifically, *DCL1* primarily generates 21 nt miRNAs, whereas *DCL3* generates 24 nt miRNAs [47]. These results indicate that most novel miRNAs identified in this study are likely processed by *DCL3*, a pattern consistent with observations in other wild rice species [48]. Mature miRNAs need to be loaded onto argonaute (ago) proteins to bind target gene mRNAs. miRNAs starting with U or A are more readily incorporated into ago proteins because these nucleotides exhibit better structural compatibility with the rigid loop of the MID (Middle) domain in Ago proteins compared to C or G [49]. In this study, the first nucleotide of known miRNAs of 20–21 nt and novel miRNAs of 20–22 nt showed a preference for U. For 24 nt miRNAs, A was the most frequent initial nucleotide in both known and novel miRNAs, whereas C showed the lowest preference (Figure 3).

The identification of miRNAs and their functional studies in an increasing number of species have laid the foundation for studying the evolution and functional diversification of miRNA families. Mature miRNAs exhibit a high degree of structural conservation during evolution, with only minor nucleotide variations observed among members of the same family [50]. In this study, we analyzed the distribution of 63 identified known miRNA families across 60 species spanning animals, plants, and microorganisms. Notably, some ancient miRNA families conserved in multicellular plants—including *MIR156*, *MIR160*, *MIR162*, *MIR164*, *MIR319/159*, *MIR393*, *MIR394*, *MIR395*, *MIR396*, *MIR397*, *MIR408*—were identified (Table 5) [40]. Their evolutionary conservation not only highlights functional preservation across plant lineages but also suggests critical regulatory roles in plant growth and development. Although some plant miRNAs exhibit high conservation, the long-term evolutionary process in plants has been accompanied by the emergence of novel tissue structures, leading to both the origin of new miRNA families and the loss of ancestral ones [41,51]. In this study, we identified several rice-specific miRNA families (e.g., *MIR439*, *MIR1319*, *MIR1428*, *MIR1437*, *MIR1846*, *MIR1861*, *MIR1862*, *MIR2121*, *MIR2863*, *MIR2871*), which may play specific biological functions in rice (Table 5). However, it should be noted that the classification of miRNA families in this study relies solely on data from 60 species in the miRbase database and literature indexed in PubMed. As miRbase does not comprehensively cover all species and miRNAs identification in different species remains an ongoing effort, coupled with the limited selection of species in this study, the classification of certain miRNA families of the last two types in Table 5 may require future revision.

### 4.2. MiRNAs Involved in the Cold Stress Response and the Cold Tolerance Regulation of Rice

Strong cold tolerance is a desirable trait in rice, enabling plants to survive under cold stress and thereby maintaining yield and grain quality. Multiple studies have demonstrated the significant role of miRNAs in regulating cold tolerance of rice [13,52]. The *MIR319* family in rice comprises two members, *osa-miR319a* and *osa-miR319b*. Transgenic rice seedlings overexpressing *osa-miR319a/b* exhibited enhanced cold tolerance (4 °C) after cold acclimation (12 °C). The expression downregulation of two target genes of *osa-miR319* (*OsPCF5* and *OsPCF8*) also enhanced the cold tolerance of plants after low-temperature acclimation [53]. The function of *miR319* in cold tolerance is evolutionarily conserved across multiple plant species [54,55]. Compared to *osa-miR319*, the highly conserved member of the *MIR156* family, *osa-miR156k*, negatively regulates cold tolerance in rice. Transgenic rice overexpressing *osa-miR156k* showed increased sensitivity to cold stress, accompanied by downregulation of its target genes (*OsSPL3*, *OsSPL14*, and *OsSPL17*) [56]. *MIR1320*, a rice-specific miRNA family, produces two mature forms (*osa-miR1320-3p* and *osa-miR1320-5p*) whose expression declines under cold stress. Overexpression of *osa-miR1320* enhanced cold tolerance, while its knockout compromised cold tolerance in transgenic plants. The transcription factor gene *OsERF096* (a member of the *APETALA2*/ethylene-responsive factor [ERF] family) is a direct target of *osa-miR1320*. The *miR1320*-*OsERF096* module regulates cold tolerance by inhibiting jasmonic acid (JA)-mediated cold signaling [57]. Furthermore, numerous miRNAs responsive to cold stress in rice have been identified in several studies [58,59].

In this study, a total of 59 differentially expressed miRNAs responding to cold treatment were identified in CLWR, while 19 were identified in 9311 (Figure 6). Among them, 8 miRNAs were found to be overlapping, namely *nov-m1941-3p*, *nov-m0505-3p*, *osa-miR319b*, *osa-miR319a-3p*, *nov-m2224-3p*, *nov-m0329-5p*, *nov-m3035-3p*, and *nov-m1139-3p* (Table 6). These shared miRNAs may represent conserved regulators of cold tolerance in rice, as their consistent response across genotypes suggests a core role in cold adaptation. Further functional validation of these candidates is warranted to elucidate their mechanisms. Among the overlapping miRNAs, *osa-miR319b* was previously reported to enhance cold tolerance in rice [53]. However, in this study, *osa-miR319b* expression was upregulated in both CLWR and 9311 under cold stress, contrasting with its reported downregulation in prior research [53]. This discrepancy may be due to variations in experimental conditions, such as the use of distinct rice varieties, sampling time points, or tissue types. Interestingly, *nov-m1941-3p* exhibited significant upregulation under cold stress in CLWR, but significant downregulation in 9311, indicating divergent regulatory roles of this miRNA in the two genotypes. MiRNAs involved in rice cold tolerance regulation, including *miR319* and *miR156*, as well as some conserved miRNAs known to be associated with cold tolerance in other species, such as *miR160* and *miR164*, exhibited significant differential expression in CLWR under cold stress [60,61].

### 4.3. The Cold-Responsive miRNAs and Their Target Genes in CLWR

Given the strong cold tolerance of CLWR compared to 9311, miRNAs displaying significant cold-responsive expression changes in CLWR—but not in 9311—or those with smaller fold changes, extremely low expression levels, or opposite patterns in 9311 are likely to be specific regulatory factors contributing to the strong cold tolerance of CLWR. As regulatory small RNAs in organisms, miRNAs generally exert their biological functions by downregulating target gene expression. Studies have shown that many target genes of miRNAs are transcription factors and kinases [13,45,52,57]. We performed target gene prediction for differentially expressed miRNAs under cold stress in CLWR and 9311. A substantial number of target genes were identified, including several major genes previously reported to regulate cold tolerance in rice (Supplementary Excel Files S5 and S6). In CLWR, the expression of *nov-m2224-3p* was significantly induced after cold treatment. Although this miRNA was also significantly upregulated in 9311, the magnitude of upregulation was much smaller compared to that in CLWR. *Nov-m2224-3p* was predicted to have 150 target genes, including many transcription factor and protein kinase genes. Among these targets, *CTB4a*, a major cold tolerance gene in rice that encodes a protein kinase, was identified. Haplotype analysis revealed that the cold-tolerant haplotype of *CTB4a* likely originated from *Oryza sativa* subsp. *japonica*. The observed differences in cold tolerance among germplasms carrying different haplotypes may be attributed to sequence variations in the promoter region of *CTB4a*, which lead to differential expression levels [5]. *Osa-miR396e-5p*, a member of the *MIR396* family, was significantly downregulated in CLWR under cold treatment. One predicted target gene of *osa-miR396e-5p* is *OsGRF4*/*LGS1*. The *miR396-GRF4* module has been reported to regulate both rice grain size and cold tolerance [42]. *Osa-miR164c* was upregulated upon cold treatment in both CLWR and 9311, but its expression level was much higher in CLWR after cold treatment. This miRNA was predicted to have 11 target genes, five of which encode NAC transcription factors. Notably, NAC transcription factors regulate cold tolerance across various species, including rice, Arabidopsis, rapeseed (*Brassica napus*), celery (*Apium graveolens*), and banana (*Musa acuminata*) [62,63]. Notably, additional differentially expressed miRNAs may play a specific role in cold tolerance regulation in CLWR. The number of differentially expressed miRNAs (Figure 6) and the functional annotation analysis of their target genes (Figure 8 and Figure S2; Supplementary Excel Files S5 and S6) also indicated that CLWR activates more extensive miRNAs and their target genes than 9311 to resist cold stress. In the future, selected differentially expressed miRNAs and their target genes can be used to create transgenic materials for cold tolerance assessment and analysis, which will help identify important miRNAs and target genes involved in rice cold tolerance regulation, providing assistance for genetic breeding research on cold tolerance in rice.

## 5. Conclusions

Cold tolerance was evaluated on seedlings of CLWR and 9311. The results indicated that CLWR possesses a strong cold tolerance. Additionally, small RNA sequencing and analyses were performed on eight samples of CLWR and 9311 before and after the cold treatment. The characteristics of small RNAs in these two varieties were revealed. There were 59 differentially expressed miRNAs in CLWR before and after cold treatment, with 19 in 9311, and an overlap of eight miRNAs between the two. A large number of target genes for these differentially expressed miRNAs were identified, including several previously reported cold tolerance genes in rice. Our results demonstrate that CLWR activates a more extensive network of miRNAs and target genes to cope with cold stress compared to 9311. Future studies should focus on validating the functions of these miRNAs and their target genes to elucidate their precise roles in regulating cold tolerance in rice.

## Figures and Tables

**Table 1:** Sample information for small RNA library construction.

Sample Name	Sample Group Name	
CLWR1	A	CLWR before cold stress treatment
CLWR2	A	
CLWR3	B	CLWR after cold stress treatment
CLWR4	B	
C9311_1	C	9311 before cold stress treatment
C9311_2	C	
C9311_3	D	9311 after cold stress treatment
C9311_4	D	

**Table 2:** The number of reads (including duplicate counts) and percentage statistical results of different small RNAs in each sample.

sRNA Type	C9311_1	C9311_2	C9311_3	C9311_4	CLWR1	CLWR2	CLWR3	CLWR4
known miRNA	228,285 (2.75%)	732,676 (6.16%)	155,152 (2.75%)	303,054 (3.49%)	236,699 (3.85%)	315,141 (3.26%)	206,688 (1.72%)	278,686 (2.53%)
novel miRNA	3967 (0.05%)	18,880 (0.16%)	2154 (0.04%)	17,100 (0.2%)	7439 (0.12%)	5909 (0.06%)	8953 (0.07%)	10,665 (0.1%)
rRNA	1,877,497 (22.6%)	2,028,028 (17.05%)	641,400 (11.36%)	2,186,266 (25.17%)	1,160,000 (18.85%)	2,367,622 (24.5%)	2,349,990 (19.51%)	1,822,779 (16.53%)
tRNA	204,779 (2.46%)	179,233 (1.51%)	153,322 (2.72%)	167,800 (1.93%)	59,386 (0.96%)	60,392 (0.62%)	371,338 (3.08%)	298,550 (2.71%)
snoRNA	0 (0.0%)	0 (0.0%)	0 (0.0%)	0 (0.0%)	0 (0.0%)	0 (0.0%)	0 (0.0%)	0 (0.0%)
snRNA	51,813 (0.62%)	31,443 (0.26%)	7527 (0.13%)	33,830 (0.39%)	27,647 (0.45%)	38,775 (0.4%)	21,052 (0.17%)	17,736 (0.16%)
repbase	74,547 (0.9%)	55,047 (0.46%)	19,989 (0.35%)	64,544 (0.74%)	50,173 (0.82%)	39,659 (0.41%)	38,147 (0.32%)	105,509 (0.96%)
exon	606,661 (7.3%)	960,817 (8.08%)	204,472 (3.62%)	658,043 (7.58%)	454,638 (7.39%)	980,238 (10.14%)	448,891 (3.73%)	478,883 (4.34%)
intron	352,405 (4.24%)	784,565 (6.6%)	190,350 (3.37%)	342,383 (3.94%)	364,626 (5.92%)	581,191 (6.01%)	350,975 (2.91%)	378,482 (3.43%)
unknown	4,909,172 (59.08%)	7,104,356 (59.73%)	4,272,048 (75.66%)	4,911,645 (56.56%)	3,794,403 (61.65%)	5,276,771 (54.59%)	8,247,072 (68.48%)	7,633,435 (69.24%)
total	8,309,126	11,895,045	5,646,414	8,684,665	6,155,011	9,665,698	12,043,106	11,024,725

**Table 3:** The numbers of known miRNA types and novel miRNA types in each sample group.

Sample	Known miRNAs	Novel miRNAs	Total
A	443	644	1087
B	433	727	1160
C	426	1348	1774
D	397	1113	1510

**Table 4:** The number of known miRNA types and novel miRNA types for different lengths from all samples.

Length of Known miRNAs (nt)	Number	Length of Novel miRNA (nt)	Number
-	-	18	1
19	2	19	1
20	126	20	232
21	827	21	396
22	178	22	322
23	8	23	488
24	225	24	2149
25	1	25	4
-	-	26	1
-	-	27	1

**Table 5:** The distribution results of known miRNA families in different species.

Distribution Type	miRNA Family Name
Present in plants	*MIR156*; *MIR159*/*319*; *MIR160*; *MIR162_2*; *MIR164*; *MIR166*; *MIR167_1*; *MIR168*; *MIR169_1*; *MIR169_2*; *MIR171_1*; *MIR171_2*; *MIR172*; *MIR390*; *MIR393*; *MIR394*; *MIR395*; *MIR396*; *MIR397*; *MIR398*; *MIR399*; *MIR408*; *MIR827*; *MIR529*; *MIR530*; *MIR535*; *MIR814*; *MIR2118*; *MIR818*; *MIR1440*
Present in Poaceae plants	*MIR1878*; *MIR2275*; *MIR437*; *MIR444*; *MIR529*; *MIR531*
Present in rice	*MIR1319*; *MIR1428*; *MIR1437*; *MIR1846*; *MIR1861*; *MIR1862*; *MIR1863*; *MIR1882*; *MIR1883*; *MIR2121*; *MIR2863*; *MIR2871*; *MIR2873*; *MIR396_2*; *MIR3980*; *MIR439*; *MIR5079*; *MIR5143*; *MIR5148*; *MIR5157*; *MIR5160*; *MIR5521*; *MIR5539*; *MIR810*; *MIR812*; *MIR815*; *MIR820*

**Table 6:** The miRNAs differentially expressed between two groups.

Comparative Groups	Up-Regulated miRNAs	Down-Regulated miRNAs
AvsB (A serves as the control group; differentially expressed in group B)	*osa-miR156h-5p*; *osa-miR3979-3p*; *miR3979-5p*; *osa-miR1861n*; *nov-m1707-5p*; *nov-m0433-3p*; *nov-m1813-5p*; nov-m1941-3p**; *nov-m0466-3p*; *nov-m2120-3p*; *osa-miR156c-5p*; *nov-m2755-5p*; *osa-miR156k*; *osa-miR156i*; *nov-m1324-3p*; *osa-miR156a*; nov-m0505-3p**; osa-miR319b**; *osa-miR156d*; osa-miR319a-3p**; *osa-miR156e*; *osa-miR164e*; nov-m2224-3p**; *osa-miR159f*; *nov-m1260-3p*; *osa-miR156f-5p*; *osa-miR156b-3p*; *osa-miR156g-5p*; nov-m3035-3p**; nov-m1139-3p**; *osa-miR156b-5p*; *osa-miR156l-5p*; *osa-miR156j-5p*; *nov-m2646-3p*; *osa-miR1859*; *nov-m2111-3p*; *nov-m2417-3p*	*nov-m1440-5p*; *nov-m0406-3p*; *osa-miR160f-5p*; *nov-m1153-3p*; *nov-m0088-3p*; *osa-miR396c-5p*; *osa-miR396f-5p*; *osa-miR164a*; *osa-miR5076*; *nov-m2954-3p*; *osa-miR172a*; *osa-miR164f*; *osa-miR164b*; *nov-m2121-3p; osa-miR172d-5p*; nov-m0329-5p**; *nov-m1983-5p*; *osa-miR396e-5p*; *osa-miR172d-3p*; *osa-miR1423-5p*; *nov-m2066-3p*; *nov-m2963-3p*
CvsD (C serves as the control group; differentially expressed in group D)	nov-m1139-3p**; nov-m0505-3p**; osa-miR319b**; *nov-m0671-5p*; *osa-miR1861h*; *osa-miR1861j*; *nov-m1964-3p*; nov-m2224-3p**; nov-m3035-3p**; *nov-m1699-5p*; *nov-m0653-3p*; *nov-m3552-5p*; osa-miR319a-3p**	*osa-miR167h-3p*; *nov-m3553-3p*; *nov-m0622-3p*; nov-m1941-3p**; *nov-m3108-3p*; nov-m0329-5p**

Note: miRNAs** represent these miRNAs exhibit differential expression in both the AvsB and CvsD comparisons.

## Data Availability

The raw reads of the 8 small RNA libraries were uploaded to the SRA database of the NCBI with the BioProject ID PRJNA1078136. All raw data used for the analyses is available at http://www.ncbi.nlm.nih.gov/bioproject/1078136, accessed on 20 February 2024. The datasets supporting the conclusions of this article are included within the article and its Supplementary files. Any other information, if required, will be made available by the corresponding author on request.

## Supplementary Materials

The following supporting information can be downloaded at: https://www.mdpi.com/article/10.3390/life15121896/s1, Figure S1: PCA. Figure S2: GO enrichment analysis (CvsD_targets). Table S1: The statistical results of sequencing data of small RNA libraries. Table S2: The expression level of 9 miRNAs in the small RNA transcriptomes. File S1: The primer information in this study; File S2: The information of all identified known miRNAs; File S3: The information of all identified novel miRNAs; File S4: The expression levels of all the identified miRNAs in transcriptomes; File S5: Target gene prediction results of differentially expressed miRNAs in CLWR (AvsB); File S6: Target gene prediction results of differentially expressed miRNAs in 9311 (CvsD); File S7: GO term clustering results of the target genes of differentially expressed miRNAs in CLWR (AvsB); File S8: GO term clustering results of the target genes of differentially expressed miRNAs in 9311 (CvsD).
