# Supplementary material for: Identification of miRNAs in Response to Cold Stress in ‘Chaling’ Common Wild Rice (Oryza rufipogon Griff.)
Source: Life (Basel). 2025 Dec 11;15(12):1896. doi: 10.3390/life15121896 (PMC12734129; doi:10.3390/life15121896)
Supplement: Supplementary file 1 [file life-15-01896-s001.zip › Table S1:The statistical results of sequencing data of small RNA libraries.pdf]

Table S1. The statistical results of sequencing data of small RNA libraries.

Sample	Raw reads	Clean reads	Q20 (%)	Useful reads (18nt-32nt)	Useful reads / clean reads (%)	Total mapped reads
C9311_1	19791677	18235769	99.61	8309126	45.56	2820984
C9311_2	19106413	16839956	99.5	11895045	70.64	4091799
C9311_3	12919474	10324944	99.52	5646414	54.69	1398400
C9311_4	14769896	14018939	99.49	8684665	61.95	2332460
CLWR1	14759237	13024627	99.64	6155011	47.26	2015442
CLWR2	17472177	15202815	99.59	9665698	63.58	3360059
CLWR3	18509992	16577271	98.53	12043106	72.65	2154301
CLWR4	19524095	16412397	99.6	11024725	67.17	3028672
