# Supplementary material for: Identification of miRNAs in Response to Cold Stress in ‘Chaling’ Common Wild Rice (Oryza rufipogon Griff.)
Source: Life (Basel). 2025 Dec 11;15(12):1896. doi: 10.3390/life15121896 (PMC12734129; doi:10.3390/life15121896)
Supplement: Supplementary file 1 [file life-15-01896-s001.zip › Table S2:The expression level of 9 miRNAs in the small RNA transcriptomes.pdf]

Table S2. The expression level of 9 miRNAs in the small RNA transcriptomes.

MiRNA name	The expression level in group A (TPM)	The expression level in group B (TPM)	The expression level in group C (TPM)	The expression level in group D (TPM)
osa-miR159f	4299.05	18507.17	9713.055	15701.85
osa-miR164c	15.075	166.205	9.585	55.17
osa-miR167h-3p	1732.29	1670.62	3621.915	813.14
osa-miR1857-5p	12.235	187.45	24.815	58.28
osa-miR1859	39.26	486.52	2.3	6.675
osa-miR1861g	835.59	973.13	2.3	6.675
osa-miR535-5p	771.7	2430.81	1028.81	1525.26
osa-miR3980a-5p	32.31	17.68	2533.795	3227.815
nov-m2224-3p	0	258.985	9.785	92.315
