# Supplementary figures and images for: Identification of miRNAs in Response to Cold Stress in ‘Chaling’ Common Wild Rice (Oryza rufipogon Griff.)
Source: Life (Basel). 2025 Dec 11;15(12):1896. doi: 10.3390/life15121896 (PMC12734129; doi:10.3390/life15121896)

# PCA

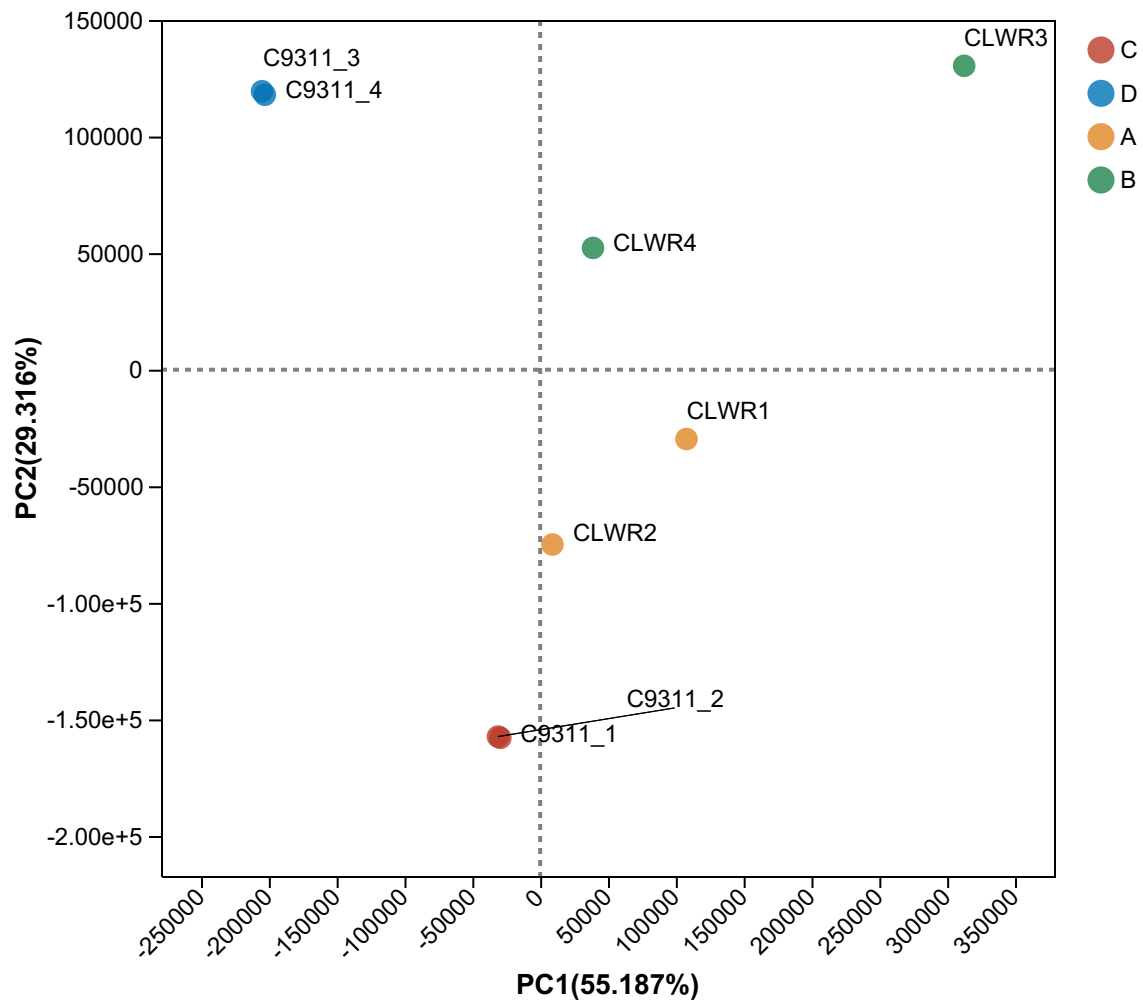

Supplement: Supplementary file 1 [file life-15-01896-s001.zip › Figure S1.pdf]

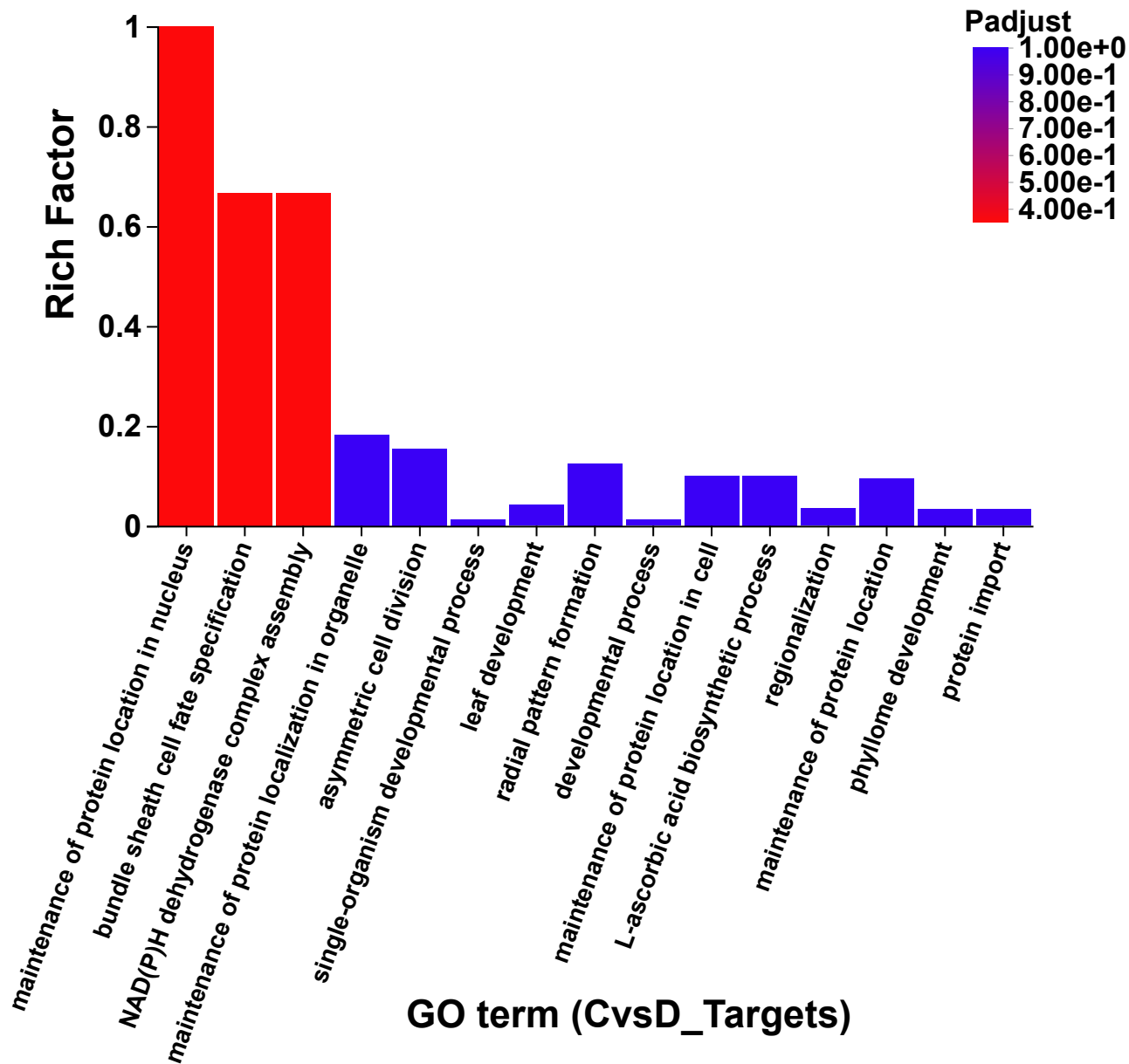

Supplement: Supplementary file 1 [file life-15-01896-s001.zip › Figure S2.pdf]
